# Developmental trajectories of sleep in offspring of parents with mood disorders

**DOI:** 10.1017/S0033291726105170

**Published:** 2026-07-29

**Authors:** Zachary Hubshman, Anna Nazarova, Mayank Ramchandani, Jason d’Eon, Nitya Adepalli, Kathryn Freeman, Max Wang, Ryan E.R. Reid, Barbara Pavlova, Rudolf Uher

**Affiliations:** 1 Dalhousie University, Canada; 2 Nova Scotia Health Authority, Canada; 3 St Francis Xavier University, Canada

**Keywords:** actigraphy, cohort, development, familial risk, mood disorders, sleep

## Abstract

**Background:**

Youth at familial risk for mood disorders often report disturbed sleep, yet it is unclear which aspects of sleep distinguish them from low-risk peers or when differences emerge developmentally.

**Methods:**

A total of 176 youth at familial risk for mood disorders (mean age = 14.6 years) and 119 control youth (mean age = 13.8 years), aged 8 to 25, wore wrist actigraphy continuously for 2 weeks. Total sleep time, sleep midpoint timing, and sleep regularity were derived from actigraphy data. Generalized additive mixed models estimated nonlinear developmental trajectories across age. Confidence bands identified age intervals at which groups diverged.

**Results:**

High- and low-risk youth followed different developmental patterns of total sleep time (*F* = 6.35, adjusted *p* < 0.001), sleep midpoint timing (*F* = 11.3, adjusted *p* < 0.001), and sleep regularity (*F* = 3.96, adjusted *p* = 0.028). High-risk youth had lower total sleep time in middle childhood (ages 8–8.8) and higher total sleep time in young adulthood (ages 18.2–23.9). They had a later sleep midpoint in late childhood (ages 10.2–14.1) and an earlier sleep midpoint from late adolescence to young adulthood (ages 17.3–25.0). High-risk youth slept more regularly from late adolescence to young adulthood (ages 17.8–21.7).

**Conclusions:**

High-risk youth showed poorer sleep than controls in early adolescence but better sleep in late adolescence through young adulthood, possibly reflecting shifting sleep priorities across development, though the mechanisms underlying these differences remain unclear.

## Introduction

Children of parents with a mood disorder, including major depressive disorder and bipolar disorder, are at an increased risk of developing a mood disorder (Uher et al., 2023). Although mood disorders typically emerge in late adolescence to early adulthood, onset often occurs in early to mid-adolescence in high-risk youth (Uher et al., 2023). Children at high risk of mood disorders frequently display mood-related symptoms, such as anxiety and irritability, consistent with early manifestations of mood vulnerability (Harrison et al., 2025; Weissman et al., 2016; Whelan, Leibenluft, Stringaris, & Barker, 2015). Sleep dysregulation is a core feature of mood disorders, including irregular sleep–wake patterns, prolonged sleep onset latency, insomnia, and circadian rhythm disruptions (Verkhratsky, Nedergaard, Steardo, & Li, 2020; Walker, Walton, DeVries, & Nelson, 2020). Sleep disturbances not only accompany existing mood disorders but also precede them, with longitudinal studies showing that disturbed sleep predicts later development of mood disorders (Alvaro, Roberts, Harris, & Bruni, 2017; Uher et al., 2024).

Previous studies comparing self-reported sleep between offspring with at least one parental diagnosis of a mood disorder (FHR) and offspring with no parental diagnosis of a mood disorder (CO) indicate that FHR youth experience greater sleep disturbances (Hamilton et al., 2020; Maoz et al., 2014). However, these findings provide limited insight into the nature of sleep disturbance, as most studies rely on global sleep scores or binary classifications of ‘sleep problems’, which obscure specific patterns of dysregulation. Only a few studies have examined specific self-reported sleep characteristics in FHR compared to CO. Levenson et al. (2015) found that FHR youth in early adolescence slept significantly earlier than CO, though no differences in sleep duration were observed. Another study by Soehner et al. (2016) found no differences in either sleep timing or sleep duration between FHR and CO youth in middle adolescence. These inconsistent findings may partly reflect the limitations of self-report, as such measures often overestimate actual sleep duration and timing, limiting their utility for estimating circadian parameters (Lauderdale et al., 2008).

Actigraphy is an objective sleep measure, which provides estimates of sleep–wake states comparable to those from polysomnography in pediatric populations (Ancoli-Israel et al., 2003; Schneider, Fárková, & Bakštein, 2022). Because actigraphy can reliably estimate sleep–wake states, it enables assessment of three key sleep domains: sleep duration, sleep timing, and regularity of sleep. To our knowledge, three studies have examined objectively measured sleep duration, timing, and regularity of sleep in youth at FHR compared to CO. For sleep duration, two studies reported no group differences in middle childhood and early adolescence (Estrada-Jaramillo et al., 2022; Sebela, Novak, Kemlink, & Goetz, 2017), whereas one study found that FHR youth slept longer than controls in late adolescence (Scott et al., 2016). For sleep timing, two studies reported no group differences in timing of sleep in early and late adolescence (Estrada-Jaramillo et al., 2022; Scott et al., 2016). Only one study has examined regularity of sleep, also reporting no differences between groups in middle childhood (Sebela, Novak, Kemlink, & Goetz, 2017). One possible explanation for the inconsistency between self-report and actigraphy findings is that the effects of familial risk on sleep may not be evident in early development but instead emerge later in adolescence or young adulthood. All previous studies relied on linear models when evaluating age-related differences in sleep, despite well-established nonlinear changes in sleep across the lifespan. For example, sleep duration is highest in childhood and declines steeply with age, while sleep timing shifts later during adolescence before advancing again in older adulthood (Colrain & Baker, 2011; Hirshkowitz et al., 2015; Li, Vitiello, & Gooneratne, 2018; Ohayon, Carskadon, Guilleminault, & Vitiello, 2004). Such approaches may lack sensitivity to detect age-specific or developmentally dynamic effects of familial risk.

One study has found FHR children slept significantly less than CO children, whereas FHR adolescents slept more than their CO peers (Sebela, Novak, Kemlink, & Goetz, 2017). While this study examined whether sleep differences between FHR and CO varied by developmental stage, it was limited by segmenting age into two periods. Developmental changes in sleep should be examined across a continuous age spectrum using nonlinear approaches to capture age-related differences at higher resolution.

To the best of our knowledge, no research has evaluated whether objectively derived sleep parameters show different developmental trajectories between FHR and CO youth. We examined developmental trajectories across three major domains of sleep: total sleep time, sleep timing, and sleep–wake patterns. We selected these domains because actigraphy provides high accuracy in identifying sleep onset and wake times, enabling reliable estimation of total sleep time, sleep timing, and sleep–wake regularity (Krystal & Edinger, 2008). Moreover, disruptions in these domains have been consistently observed in individuals with mood disorders (Murphy & Peterson, 2015). We assessed whether age-related changes in total sleep time, sleep midpoint timing, and sleep regularity differed between FHR and CO. We expected FHR youth to show more extreme total sleep time (either higher or lower), later sleep midpoint, and a lower sleep regularity as they aged.

## Methods

### Participants

Participants aged 8–25 from the Families Overcoming Risks and Building Opportunities for Wellbeing cohort (Uher et al., 2014) were invited to participate in the actigraphy study. We included participants with a parental diagnosis of major depressive disorder, bipolar disorder, or no parental diagnosis of mood disorders. We excluded youth with parental diagnosis of schizophrenia or non-affective psychotic disorder. Additionally, participants with fewer than three weekday nights or fewer than one weekend night of actigraphy were excluded to improve the reliability and stability of derived sleep metrics (Lee, 2024). All participants and their parents or legal guardians signed informed consent. The study was approved by the Research Ethics Board of the Nova Scotia Health Authority.

### Family history

Assessors completed a Structured Clinical Interview for the Diagnostic and Statistical Manual of Mental Disorders, 5th edition (DSM-5) with each parent to determine familial risk (First, 2015). We classified participants as belonging to the FHR group if at least one biological parent met diagnostic criteria for major depressive disorder or bipolar disorder, type I or II, according to the DSM-5. We classified participants as comparison offspring (CO) if neither of their biological parents met criteria for a major mood or psychotic disorder.

### Actigraphy

Participants wore an actigraph on their non-dominant hand for 2 weeks. MotionLogger® uniaxial piezoelectric actigraphs were used from May 2015 to March 2021. Motion is recorded through movement at a rate of 16 times per second and stored as summary activity counts per minute. GENEActiv® triaxial Micro-Electro-Mechanical System accelerometers were used from July 2021 to December 2025. Triaxial motion is recorded by measuring acceleration 50 times per second and storing the complete raw data.

### Sleep measures

To cover key elements of sleep and circadian rhythm relevant to psychopathology, we selected indices reflecting total sleep time, timing of sleep within the circadian period, and night-to-night regularity of sleep and wakefulness. Data from each of the accelerometers went through separate processes.

Observations collected using the Motionlogger® device derived sleep measures through ActionW 2.7 software. Within ActionW, sleep–wake classification was performed using the Cole–Kripke algorithm, which applies weighted activity counts to determine sleep–wake status across epochs. Observations that used GENEActiv® derived sleep measures through GGIR version 3.3–4, an open-source R package designed to analyze multi-day accelerometer values (van Hees & Migueles, 2025). After removing non-wear periods, detected as extended periods of lack of acceleration in all directions (van Hees et al., 2013), we determined the sleep window timing by the Heuristic Distribution of Change in Z-Axis detection algorithm (HDCZA), which identifies the longest block of no changes in the vertical direction.

#### Total sleep time

Total sleep time (TST) represents the total duration of time that an individual spends inactive during the sleep window. TST was calculated in hours as the total sleep window duration minus the time spent active within that window.

#### Sleep midpoint

Sleep midpoint is a continuous measure that reflects an individual’s sleep timing. It is the midpoint between sleep onset and wake time. Sleep midpoint was calculated as wake time minus half the total sleep window duration and expressed as hours from midnight.

#### Sleep regularity index

Sleep regularity index is a continuous measure ranging from −100 to +100 that quantifies the probability that the sleep–wake state is consistent across consecutive days. We calculated sleep regularity indexes by estimating the percentage of minutes in the asleep–awake state that are identical over two successive days (Phillips et al., 2017).

### Data analysis

We fit three generalized additive mixed models (GAMMs) to examine nonlinear age-related changes in total sleep time, sleep midpoint, and sleep regularity. Given established evidence that sleep develops nonlinearly across childhood and adolescence, GAMMs were used to model these trajectories flexibly and to assess whether developmental patterns differed between groups (Hirshkowitz et al., 2015). This approach allowed the shape of the developmental trajectory to be estimated without prior assumptions about where these changes might occur. To evaluate whether GAMMs provided a better fit than linear mixed-effects models, we compared Bayesian Information Criterion (BIC) values from linear mixed-effects models and GAMMs. A lower BIC in the GAMM indicates that the nonlinear age trajectory improved model fit beyond what was captured by a linear term, accounting for the additional model complexity.

In our analyses, a day was defined as a 24-hour period from 12:00 PM on one date to 12:00 PM on the following date. Days were considered valid if they contained at least 16 hours of detected wear time. Each valid day of sleep data constituted one observation. All models included random intercepts for individuals nested within families to account for repeated observations and family clustering. Fixed-effect covariates included sex and weekend versus weekday measurement. The linear mixed-effects model included an age-by-familial risk interaction. In generalized additive mixed models, age was modeled as a smooth term, allowing the relationship between age and the outcome to follow a flexible curve. Smooth terms were constructed using five basis functions, with separate smooths for FHR and CO to enable comparison of developmental trajectories. The *p*-values for differences between smooths were adjusted for multiple comparisons using the Benjamin–Hochberg correction.

To identify ages at which developmental trajectories diverged between FHR and CO youth, we estimated the difference between group-specific age-smooths and constructed 95% simultaneous confidence bands around this difference. Simultaneous confidence bands allow us to determine when and by how much trajectories differ, enabling interpretation of age-specific nonlinear differences while accounting for multiple comparisons across development. Age intervals where the confidence bands excluded zero were interpreted as evidence of divergence between familial high-risk and comparison youth. All analyses were performed using RStudio Version 4.4.1, and R packages *mgcv* and *gratia* (Simpson, 2024; Wood, 2017).

## Results

### Participants

Youth aged 8 to 25 were invited to participate in the actigraphy study. Over two-thirds of eligible participants consented, yielding a sample of 295 youth. 59% of participants were at familial risk for mood disorders (*n* = 176; mean age [SD] = 14.65 [3.7]). One-third of participants had no familial risk for mood disorders (*n* = 119; mean age [SD] = 13.82 [3.6]). FHR participants included 122 youth with a parental diagnosis of major depressive disorder and 54 youth with a parental diagnosis of bipolar disorder (Table 1). A chi-square test indicated that nonparticipants (*n* = 168) did not differ from participants (*n* = 295) in sex and familial risk status. An independent-samples t-test indicated that non-participators did not differ in age from participants (Supplementary Table 1).

**Table 1.** Participant characteristics Table 1. long description.

Variable	CO	FHR	Statistic	*p*-value
n	119	176		
Age (M ± SD)^a^	13.82 (3.6)	14.65 (3.7)	*t*(260) = −1.9	0.056
Female N (%)^b^	58 (48.7%)	81 (46.0%)	χ^2^(1) = 0.11	0.73
Race (N)^c^				0.02
White	104	160		
Black	0	5		
Asian	2	0		
First nation	0	1		
Other	13	10		

*Note*: CO, comparison offspring; FHR, familial high-risk for mood disorders.

a Independent samples *t*-test.

b Chi-square test of independence.

c Fisher’s exact test.

### Trajectories of sleep parameters

#### Model comparison

First, we examined whether sleep parameters changed linearly or nonlinearly with age. Across total sleep time and midpoint, nonlinear models demonstrated superior fit, as indicated by lower BIC values. Nonlinear models provided lower BIC for total sleep time (19,920 vs. 19,945) and midpoint (21,060 vs. 20,893), with higher BIC for sleep regularity (40,484 vs. 40,479). We report primary results through nonlinear models due to their superior or similar fit Table 2. Results from linear models are shown in Supplementary Table 2.

**Table 2.** Results Table 2. long description.

Linear terms	Total sleep time model Beta (95% CI)	Sleep midpoint modelBeta (95% CI)	Sleep regularity modelBeta (95% CI)
Intercept	7.37 (7.23, 7.51)***	3.38 (3.21, 3.56)***	84.07 (82.87, 85.28)***
FHR (group)	0.08 (−0.08, 0.23)	−0.09 (−0.28, 0.11)	−0.22 (−1.57, 1.12)
Sex (female)	0.35 (0.17, 0.53)***	0.04 (−0.19, 0.26)	−0.34 (−1.82, 1.13)
Weekend	0.08 (0.01, 0.15)*	0.48 (0.41, 0.56)***	−4.68 (−5.54, −3.81)***
Smooth terms	F (edf)	F (edf)	F (edf)
Smooth (age)	104.56 (2.32)***	85.80 (3.69)***	48.62 (2.92)***
Smooth (Age) × FHR	6.35 (3.75)***	11.30 (2.82)***	3.74 (3.21)*

*Note*: Values are reported as β (95% confidence interval). For smooth terms, values are reported as F statistics with effective degrees of freedom (edf) in parentheses. *p < 0.05, **p < 0.01, ***p < 0.001. CI = confidence intervals; edf = effective degrees of freedom; FHR = familial high-risk for mood disorders.

#### Total sleep time

**Figure 1.**
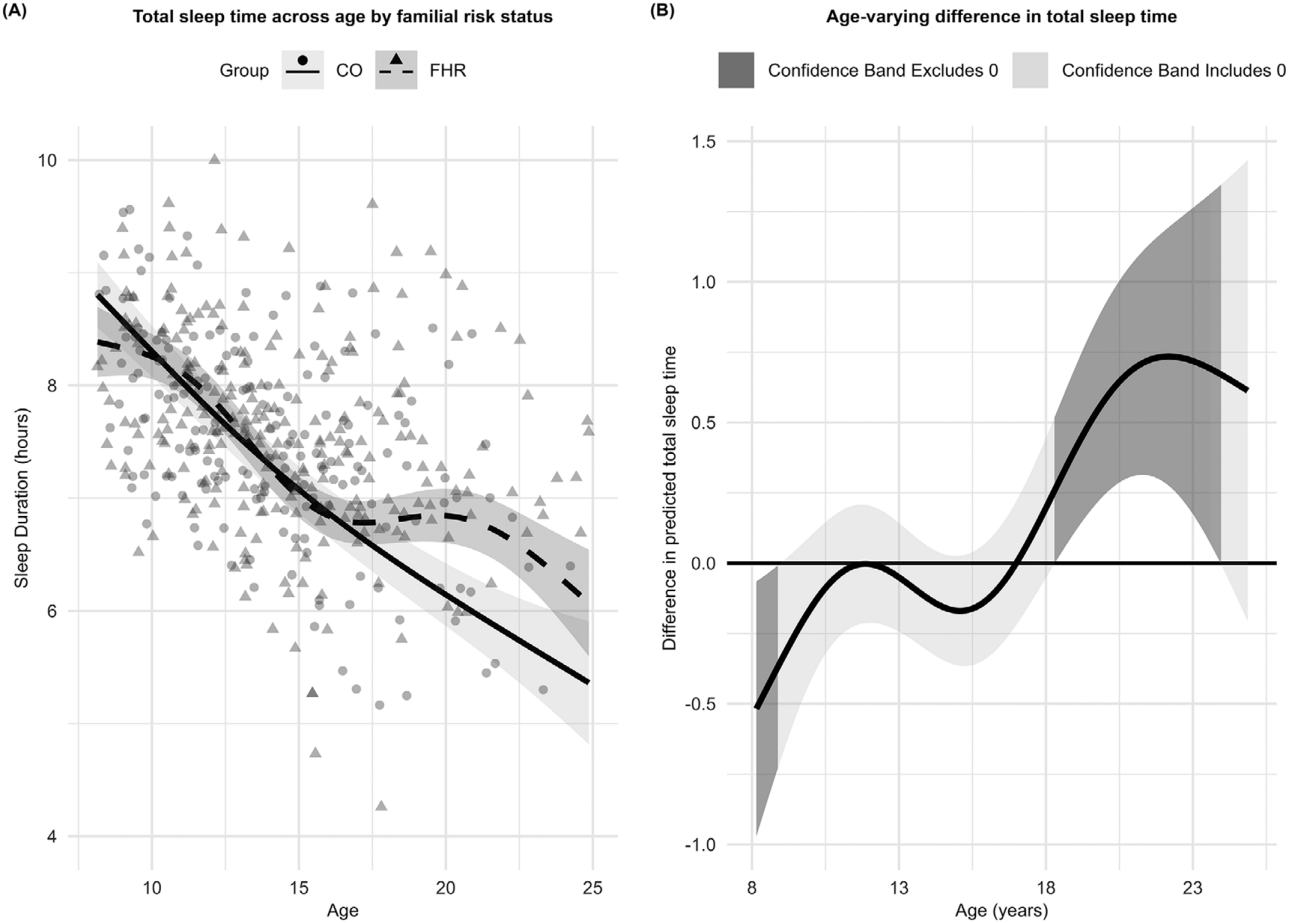
Total sleep time trajectories by familial risk status. (a) Observed mean sleep regularity across a 2-week period for each participant, plotted by age, with fitted GAMM trajectories for FHR (triangles) and CO (circles). Shaded regions represent the 95% confidence intervals of the model fits. The *p*-value corresponds to the global test of the group-by-age interaction, indicating whether overall age-dependent trajectories differ between groups. (b) Age-varying difference in total sleep time between familial high-risk and comparison offspring. The solid line is the generalized additive model estimate of the difference. Shaded regions show the simultaneous 95% confidence bands. The dark gray shading identifies the age range in which the confidence band excludes zero, indicating divergence between FHR and CO trajectories. Figure 1. long description.

Youth at familial risk for mood disorders differed from controls in the developmental trajectory of total sleep time (*F* = 6.35, adjusted *p* < 0.001). From ages 8 to 8.88, the simultaneous confidence band for the difference smooth excluded zero, indicating lower total sleep time in the FHR group than in controls. From ages 18.29 to 23.96, the simultaneous confidence band for the difference smooth excluded zero, indicating higher total sleep time in the FHR group than in controls (Figure 1).

#### Sleep midpoint

**Figure 2.**
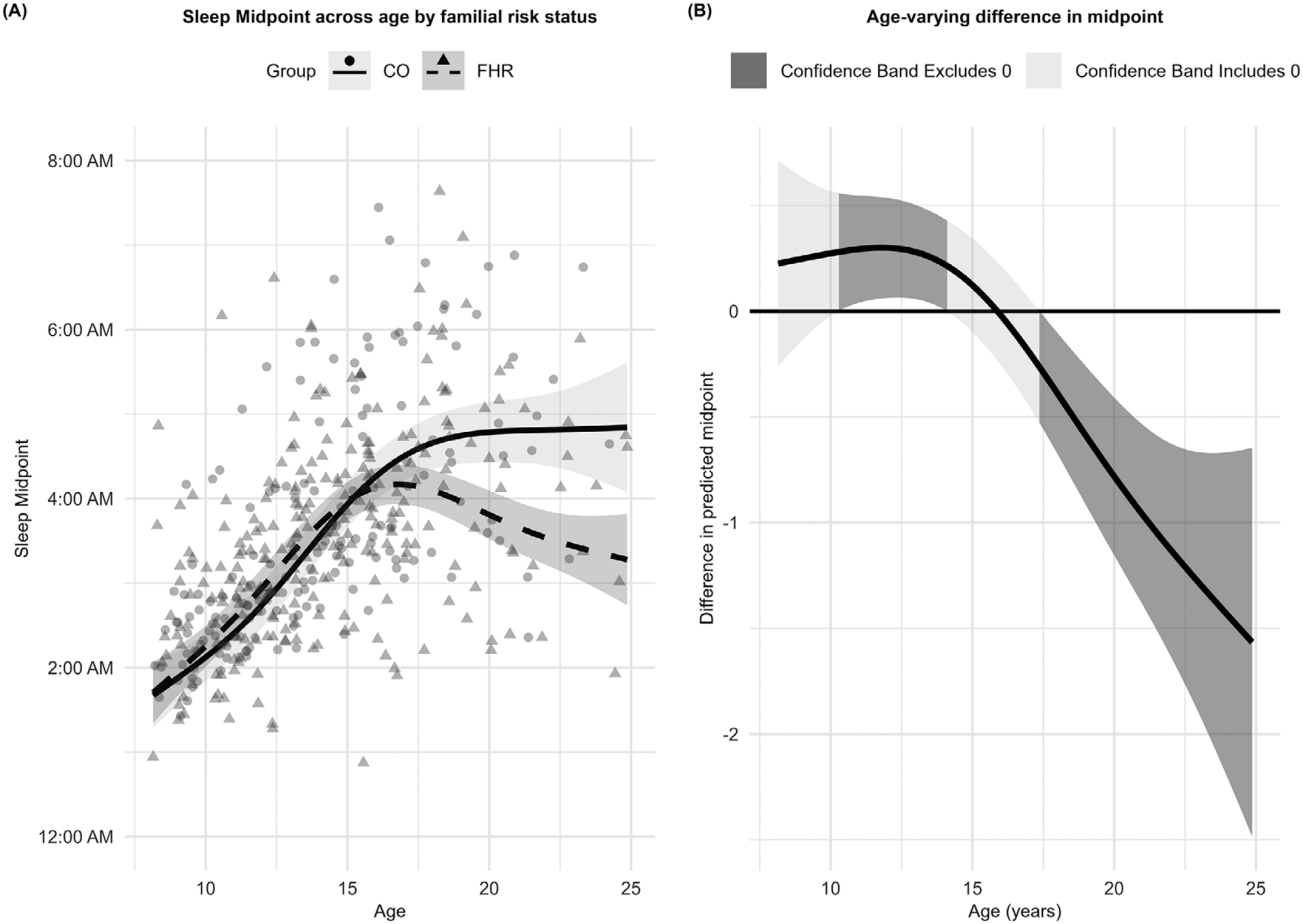
Sleep midpoint trajectories by familial risk status. (a) Observed mean sleep regularity across a 2-week period for each participant plotted by age, with fitted GAMM trajectories for FHR (triangles) and CO (circles). Shaded regions represent the 95% confidence intervals of the model fits. The *p*-value corresponds to the global test of the group-by-age interaction, indicating whether overall age-dependent trajectories differ between groups. (b) Age-varying difference in sleep midpoint between familial high-risk and comparison offspring. The solid line is the generalized additive model estimate of the difference. Shaded regions show the simultaneous 95% confidence bands. The dark gray shading identifies the age range in which the confidence band excludes zero, indicating divergence between FHR and CO trajectories. Figure 2. long description.

Youth at familial risk for mood disorders differed from controls in the developmental trajectory of sleep midpoint (*F* = 11.3, adjusted *p* < 0.001). From ages 10.22 to 14.11, the simultaneous confidence band for the difference smooth excluded zero, indicating a later sleep midpoint in the FHR group than in controls. From ages 17.36 to 25.0, the simultaneous confidence band for the difference smooth excluded zero, indicating an earlier sleep midpoint in the FHR group than in controls (Figure 2).

#### Sleep regularity index

**Figure 3.**
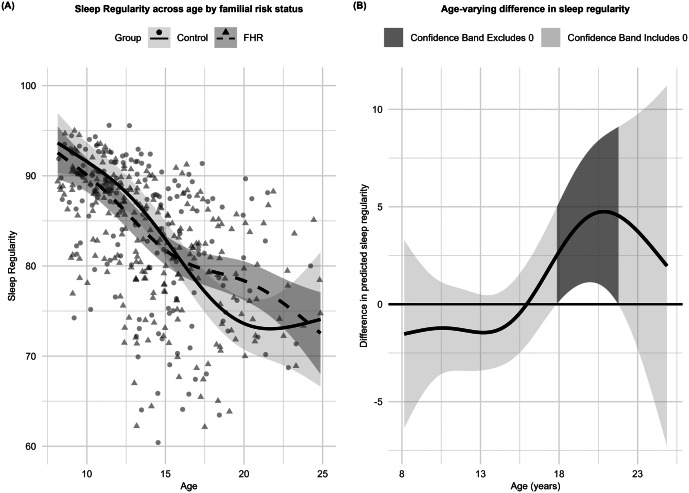
Sleep regularity trajectories by familial risk status. (a) Observed mean sleep regularity across a 2-week period for each participant plotted by age, with fitted GAMM trajectories for FHR (triangles) and CO (circles). Shaded regions represent the 95% confidence intervals of the model fits. The *p*-value corresponds to the global test of the group-by-age interaction, indicating whether overall age-dependent trajectories differ between groups. (b) Age-varying difference in sleep regularity between familial high-risk and comparison offspring. The solid line is the generalized additive model estimate of the difference. Shaded regions show the simultaneous 95% confidence bands. The dark gray shading identifies the age range in which the confidence band excludes zero, indicating divergence between FHR and CO trajectories. Figure 3. long description.

Youth at familial risk for mood disorders differed from controls in the developmental trajectory of sleep regularity (*F* = 3.73, adjusted *p* = 0.028). From ages 17.82 to 21.78, the simultaneous confidence band for the difference smooth excluded zero, indicating higher sleep regularity in the FHR group than in controls (Figure 3).

## Discussion

This cohort study found that, relative to controls, familial risk for major mood disorders was associated with different age-related trajectories of objectively measured total sleep time, midpoint timing, and sleep regularity.

We found that high-risk youth slept less in middle childhood (ages 8–9) and slept longer during young adulthood (ages 18–24). In contrast with our findings, a previous study by Estrada-Jaramillo et al. (2022) reported significantly shorter sleep duration in high-risk youth; the mean age of 14 in that study falls within an interval in which we did not observe differences by family history. Sebela, Novak, Kemlink, and Goetz (2017) found no group differences in sleep duration; the mean age of 12 in that study is consistent with our findings at earlier developmental stages, whereas Scott et al. (2016) reported longer sleep in high-risk youth; the mean age of 19 in that study aligns within an interval in which we observed longer total sleep time in FHR. Short sleep has been linked to increased all-cause mortality and suicidal ideation (Goodwin & Marusic, 2008; Windred et al., 2024). Taking age into consideration, our findings are consistent with previous research and suggest that high-risk youth may develop a healthier sleep duration in young adulthood compared with control youth.

We found that high-risk youth slept later during middle childhood to early adolescence (ages 10–14). This changed later in life where sleep occurred earlier in high-risk participants from late adolescence to young adulthood (ages 17–25). Estrada-Jaramillo et al. (2022) previously found no group differences in sleep onset time among high-risk youth; the mean age of 14 years in their study aligns with an interval in which no differences in sleep timing by family history were found. Scott et al. (2016) reported no group differences in sleep onset or offset in high-risk youth but did find earlier sleep timing. This study is consistent with our high-risk participants, who also had earlier sleep at this age. A later sleep midpoint has been associated with adverse health outcomes, including elevated cardiometabolic risk, lower physical activity, and poorer mental health during adolescence (Brombach et al., 2025; Chaput et al., 2020). Our findings validate previous research that indicated that high-risk youth may develop healthier sleep timing in young adulthood.

We found that high-risk youth have more consistent sleep in late adolescence to early adulthood (ages 18–22). Irregular sleep has been linked to an increase in all-cause mortality and depressive symptoms (Messman et al., 2024; Windred et al., 2024). Sebela, Novak, Kemlink, and Goetz (2017) found similar regularity of sleep across 12-year-old participants measured through interdaily stability, which captures day-to-day consistency in sleep–wake patterns. This is consistent with our results, which showed similar sleep regularity between high-familial-risk and control participants at earlier developmental stages. Our findings validate a previous study showing that high-risk participants may develop a more consistent sleep–wake cycle than control participants in young adulthood.

We found that healthier sleep patterns in FHR youth emerge in late adolescence across total sleep time, midpoint, and regularity relative to controls. These findings are consistent with Sebela, Novak, Kemlink, and Goetz (2017), whose early adolescent sample falls within developmental periods in which we similarly observed no group differences. In contrast, Scott et al. (2016) and Estrada-Jaramillo et al. (2022) middle to late adolescent samples reported findings that differ from our sleep duration and sleep time results at comparable ages, which may reflect their smaller sample sizes and the use of different statistical approaches. Youth at familial high-risk are more likely to develop mood disorders during the developmental period, which are commonly preceded by prodromal sleep disturbances. Moreover, since high-risk youth typically develop depressive symptoms earlier than low-risk youth, we would expect greater, not reduced, sleep disruption.

Differences may appear in late adolescence due to education and employment. A meta-analysis examining the relationship between adolescent health and later life outcomes found that youth who experience mental health problems are more likely to be unemployed and less likely to pursue post-secondary education (Hale, Bevilacqua, & Viner, 2015). Evidence from the Canadian Community Health Survey shows that unemployed adults sleep 40 minutes longer than employed adults (Wang et al., 2022). Group differences in sleep that emerge in late adolescence may reflect educational and employment engagement, since FHR participants may have fewer obligations than controls. Future research should seek to replicate these age-related patterns in FHR youth and identify the factors driving poorer sleep in middle childhood and better sleep in late adolescence. Understanding what underlies poorer sleep quality in early development could highlight useful targets for early intervention. Additionally, identifying the factors associated with healthier sleep trajectories in late adolescence may offer insight into high-risk youth.

### Strengths

Our sample size provides statistical power to detect group differences and developmental effects. The use of generalized additive models allowed us to flexibly capture the nonlinear relationship between age and sleep across development. Additionally, the construction of simultaneous confidence bands enabled estimation of age-specific group differences while controlling for multiple comparisons across age.

### Limitations

Notable limitations of this study include the lack of contextual information about participants during the sleep assessment period. We were unable to account for day-to-day factors that may influence sleep, such as daily medication use or daily behaviors, as sleep diaries were not collected in all participants. As such, unmeasured daily factors may partially or fully explain the observed differences between FHR and CO participants. Sleep trends largely emerge later in life in our sample, whereas most of our data were restricted to adolescence. Larger studies spanning late adolescence to young adulthood will be needed to clarify the effect of familial risk on sleep.

Another limitation is the use of different actigraphs. Motionlogger devices are less granular than the raw data captured by GeneActiv accelerometers. As a result, the GeneActiv data allow for non-wear detection and more refined sleep detection algorithms. Additionally, ActionW 2.7 software has limited non-wear detection, increasing the potential for misclassification of inactivity as sleep. Because we used two different sleep detection algorithms across devices, minor variability in derived sleep estimates may have been introduced. Although HDCZA and the Cole–Kripke method have demonstrated comparable validity (Chen et al., 2026), differences in their underlying scoring approaches could contribute to small discrepancies across devices.

## Conclusion

Youth with familial risk for mood disorders followed different age-related sleep trajectories compared to control youth. Specifically, high-risk youth demonstrated better sleep habits in late adolescence to young adulthood across all three domains: total sleep time, sleep timing, and sleep regularity. During childhood to early adolescence, high-risk youth showed worse sleep habits across sleep timing. These findings may reflect differences in post-secondary educational and employment contexts, as high-risk youth may have greater opportunities for sleep during early adulthood than low-risk youth.

## Supporting information

10.1017/S0033291726105170.sm001 Hubshman et al. supplementary material Hubshman et al. supplementary material

## Data Availability

Data analyzed during this study are not available. The analysis pipeline is available on Github (https://github.com/zachary-hubshman/Developmental-Trajectories-of-Sleep-in-Offspring-of-Parents-with-Mood-Disorders).

## Supplementary material

The supplementary material for this article can be found at http://doi.org/10.1017/S0033291726105170.

## Long descriptions

### Figure 1. Long description

A two-panel figure labeled A and B.

Panel A, titled Total sleep time across age by familial risk status, is a scatter plot with fitted trajectories. The x-axis is Age ranging from 10 to 25. The y-axis is Sleep Duration in hours ranging from 4 to 10.

* C O group data is represented by circles and a solid black line. The trajectory shows a steady linear decline from approximately 9 hours at age 8 to 5.5 hours at age 25.

* F H R group data is represented by triangles and a dashed black line. This trajectory starts lower than C O at age 8, around 8.5 hours, declines until age 15, plateaus between ages 15 and 20, and then declines again toward age 25.

* Both lines are surrounded by light gray shaded 95 percent confidence intervals.

Panel B, titled Age-varying difference in total sleep time, shows the calculated difference between the two groups. The x-axis is Age in years from 8 to 23. The y-axis is Difference in predicted total sleep time from -1.0 to 1.5.

* A horizontal zero line represents no difference.

* A solid black curve oscillates around the zero line until age 18, after which it rises sharply, peaking near 0.75 around age 22.

* Shaded regions represent 95 percent confidence bands. Light gray indicates the band includes zero. Dark gray indicates the band excludes zero, which occurs at the very beginning of the age range (around age 8) and significantly from age 18 to 23, where the F H R group shows higher predicted sleep time than the C O group.

Navigate back to Figure 1..

### Figure 2. Long description

A two-panel figure labeled A and B.

Panel A, titled Sleep Midpoint across age by familial risk status, features a scatter plot with fitted G A M M trajectories. The x-axis represents Age from 8 to 25. The y-axis represents Sleep Midpoint from 12:00 AM to 8:00 AM.

* C O group data is shown as circles with a solid black trend line. The line shows a steep linear increase from 2:00 AM at age 8 to 4:00 AM at age 15, then plateaus near 5:00 AM by age 25.

* F H R group data is shown as triangles with a dashed black trend line. This line follows the C O trajectory until age 15, then declines toward 3:30 AM by age 25.

* Light gray shaded regions around both lines represent 95 percent confidence intervals.

Panel B, titled Age-varying difference in midpoint, shows the calculated difference between groups. The x-axis is Age (years) from 10 to 25. The y-axis is Difference in predicted midpoint from -2 to 0.

* A horizontal zero line represents no difference.

* A solid black curve starts slightly above zero at age 8, peaks around age 12, crosses zero at age 16, and drops sharply to nearly -1.5 by age 25.

* Shaded confidence bands surround the curve. Light gray shading indicates where the band includes zero. Dark gray shading, indicating statistical divergence, appears between ages 10 to 14 and from age 17 to 25.

Navigate back to Figure 2..

### Figure 3. Long description

Panel A, titled Sleep Regularity across age by familial risk status, features a scatter plot with two fitted trend lines. The x-axis is Age ranging from 10 to 25. The y-axis is Sleep Regularity ranging from 60 to 100. Control participants are represented by circles and a solid black line. F H R participants are represented by triangles and a dashed black line. Both groups show a downward trend in sleep regularity as age increases. The solid line for the control group shows a steeper decline between ages 15 and 20 compared to the F H R group. Shaded gray regions around the lines indicate 95 percent confidence intervals.

Panel B, titled Age-varying difference in sleep regularity, shows a single black curve representing the difference in predicted sleep regularity between the two groups. The x-axis is Age years from 8 to 23. The y-axis is Difference in predicted sleep regularity from negative 5 to 10. A horizontal zero line indicates no difference. The curve starts slightly below zero, crosses the zero line around age 16, and peaks near age 21. A light gray shaded region represents the confidence band. A dark gray vertical band between ages 18 and 22 highlights the region where the confidence band excludes zero, indicating a statistically significant divergence where the F H R group has higher predicted sleep regularity than the control group.

Navigate back to Figure 3..

### Table 1. Long description

The table consists of five columns: Variable, C O, F H R, Statistic, and p-value.

* The sample size n is 119 for C O and 176 for F H R.

* Age (Mean plus or minus S D): C O is 13.82 (3.6), F H R is 14.65 (3.7). Statistic t(260) = -1.9, p-value 0.056.

* Female N (percentage): C O is 58 (48.7 percent), F H R is 81 (46.0 percent). Statistic chi-squared(1) = 0.11, p-value 0.73.

* Race (N): The p-value for the distribution is 0.02.

* White: C O is 104, F H R is 160.

* Black: C O is 0, F H R is 5.

* Asian: C O is 2, F H R is 0.

* First nation: C O is 0, F H R is 1.

* Other: C O is 13, F H R is 10.

Note: C O stands for comparison offspring and F H R stands for familial high-risk for mood disorders.

Navigate back to Table 1..

### Table 2. Long description

The table consists of four columns: Linear terms, Total sleep time model Beta 95 percent C I, Sleep midpoint model Beta 95 percent C I, and Sleep regularity model Beta 95 percent C I.

Linear terms section:

- Intercept: Total sleep time 7.37 (7.23, 7.51); Sleep midpoint 3.38 (3.21, 3.56); Sleep regularity 84.07 (82.87, 85.28). All significant at p < 0.001.

- F H R group: Total sleep time 0.08 (-0.08, 0.23); Sleep midpoint -0.09 (-0.28, 0.11); Sleep regularity -0.22 (-1.57, 1.12).

- Sex female: Total sleep time 0.35 (0.17, 0.53) p < 0.001; Sleep midpoint 0.04 (-0.19, 0.26); Sleep regularity -0.34 (-1.82, 1.13).

- Weekend: Total sleep time 0.08 (0.01, 0.15) p < 0.05; Sleep midpoint 0.48 (0.41, 0.56) p < 0.001; Sleep regularity -4.68 (-5.54, -3.81) p < 0.001.

Smooth terms section (reported as F statistics with effective degrees of freedom in parentheses):

- Smooth age: Total sleep time 104.56 (2.32); Sleep midpoint 85.80 (3.69); Sleep regularity 48.62 (2.92). All significant at p < 0.001.

- Smooth Age times F H R: Total sleep time 6.35 (3.75) p < 0.001; Sleep midpoint 11.30 (2.82) p < 0.001; Sleep regularity 3.74 (3.21) p < 0.05.

Navigate back to Table 2..
