# Supplementary material for: Developmental trajectories of sleep in offspring of parents with mood disorders
Source: Psychol Med. 2026 Jul 29;56:e224. doi: 10.1017/S0033291726105170 (PMC13439250; doi:10.1017/S0033291726105170)
Supplement: Supplementary file 1 [file S0033291726105170sup001.zip › Supplementary Document 1.docx]

**Supplementary Document 1.** Non-participation and Filtered Data

Of the 168 non-participators, 44 consented to participate but did not provide a minimum of 5 days of valid actigraphy data. The remaining 124 either did not consent to future contact or declined to participate in the sleep study. Of the 295 participants included in the study, a total of 6,229 nights of sleep data were observed, of which 5,906 (94.8%) met the minimum wear time threshold of 16 hours and were retained for analysis. Information on demographic comparison between participants and non-participators can be found in **Table S1.**

**Table S1**

| **Variable** | **Participants** | **Nonparticipants** | **Statistic** | **p** |
| --- | --- | --- | --- | --- |
| Sex (Male/Female)^a^ | 156 / 139 | 91 / 77 | χ²(1) = 0.03 | 0.86 |
| Familial risk group (CO/MDD/BP)^a^ | 119 / 122 / 54 | 65 / 59 / 40 | χ²(2) = 2.69 | 0.26 |
| Age (sd)^b^ | 14.32 (3.67) | 15.09 (4.44) | t(296) = 1.93 | 0.055 |

a. *Chi-square test of independence*
b. *Independent samples t-test.*
