# Supplementary material for: Developmental trajectories of sleep in offspring of parents with mood disorders
Source: Psychol Med. 2026 Jul 29;56:e224. doi: 10.1017/S0033291726105170 (PMC13439250; doi:10.1017/S0033291726105170)
Supplement: Supplementary file 1 [file S0033291726105170sup001.zip › Supplementary Document 2.docx]

**Supplementary Document 2.** Linear Model of Main Analysis

**Table S2**

| **Variable** | **Total Sleep Time Model**  **Beta (95% CI)** | **Sleep Midpoint Model**  **Beta (95% CI)** | **Sleep Regularity Model**  **Beta (95% CI)** |
| --- | --- | --- | --- |
| Intercept | 10.43*** [10.00, 10.87] | 0.42* [0.08, 0.76] | 104.89*** [102.05, 107.72] |
| FHR | -0.80** [-1.36, -0.24] | 1.08*** [0.62, 1.55] | -5.14* [-9.08, -1.20] |
| Age | -0.22*** [-0.25, -0.19] | 0.21*** [0.19, 0.23] | -1.45*** [-1.64, -1.27] |
| FHR × Age | 0.06*** [0.03, 0.10] | -0.08*** [-0.11, -0.05] | 0.34** [0.08, 0.60] |
| Sex (Female) | 0.38*** [0.20, 0.56] | 0.01 [-0.22, 0.24] | -0.34 [-1.80, 1.12] |
| Weekend | 0.08* [0.01, 0.15] | 0.48*** [0.41, 0.56] | -4.68*** [-5.54, -3.81] |

*Note. Values are reported as β (95% confidence interval). * p < .05, ** p < .01, *** p < .001.*
