# Supplementary material for: Developmental trajectories of sleep in offspring of parents with mood disorders
Source: Psychol Med. 2026 Jul 29;56:e224. doi: 10.1017/S0033291726105170 (PMC13439250; doi:10.1017/S0033291726105170)
Supplement: Supplementary file 1 [file S0033291726105170sup001.zip › Supplementary Document 3.docx]

**Supplementary Document 3.** Sensitivity Analysis Including Additional Covariates

All primary models were rerun adjusting for past-year mood disorder diagnosis, actigraphy device type (MotionLogger vs. GENEActiv), and participant race. Mood disorder diagnoses were derived from the Kiddie Schedule for Affective Disorders and Schizophrenia (K-SADS) using DSM-IV criteria and the Structured Clinical Interview for DSM-5 (SCID) using DSM-5 criteria, administered by trained interviewers and confirmed by consensus review with a licensed psychiatrist. Mood disorder diagnosis types are detailed in **Supplementary Table 3**, sensitivity analysis model results are presented in **Supplementary Table 4**, and corresponding developmental trajectories are displayed in **Supplementary Figures 1–3**.

**Table S3**

| **Variable** | **n (%)** | |
| --- | --- | --- |
| Past Year Major Depressive Episode | | 31 (10.5%) |
| Past Year Depressive  Disorder | | 37 (12.5%) |
| Past Year Bipolar  Disorder | | 3 (1.0%) |

**Table S4**

| **Variable** | **Sleep Midpoint Model**  **Beta (95% CI)** | **Total Sleep Time Model**  **Beta (95% CI)** | **Sleep Regularity Model**  **Beta (95% CI)** |
| --- | --- | --- | --- |
| Intercept | 3.11 (2.91, 3.32)*** | 7.90 (7.75, 8.04)*** | 86.61 (85.12, 88.10)*** |
| FHR (Group) | -0.04 (-0.24, 0.16) | 0.03 (-0.10, 0.16) | -0.54 (-1.89, 0.81) |
| Sex (Female) | 0.04 (-0.18, 0.27) | 0.32 (0.17, 0.47)*** | -0.39 (-1.85, 1.06) |
| Weekend | 0.48 (0.41, 0.56)*** | 0.08 (0.01, 0.15)* | -4.67 (-5.53, -3.81)*** |
| Geneactiv Watch | 0.33 (0.19, 0.48)*** | -0.77 (-0.89, -0.66)*** | -3.65 (-4.94, -2.36)*** |
| Past Year Mood  Disorder | 0.11 (-0.13, 0.34) | 0.02 (-0.18, 0.21) | -0.77 (-2.86, 1.33) |
| Black | 0.61 (-0.26, 1.49) | -0.57 (-1.18, 0.03). | -2.20 (-7.89, 3.49) |
| Asian | 1.63 (-0.12, 3.38). | 0.68 (-0.47, 1.84) | -6.86 (-19.59, 5.87) |
| First Nation | -0.70 (-2.75, 1.35) | -0.07 (-1.42, 1.28) | 6.79 (-6.50, 20.07) |
| Other | 0.16 (-0.30, 0.62) | -0.04 (-0.35, 0.27) | 0.35 (-2.73, 3.44) |
| **Smooth Terms** |  |  |  |
| Smooth(Age) | 51.78 (edf=3.67)*** | 42.45 (edf=2.08)*** | 34.20 (edf=2.68)*** |
| Smooth(Age) × FHR | 10.33 (edf=2.93)*** | 5.45 (edf=3.68)*** | 2.49 (edf=3.01). |

**Figure S1**

(A) Observed mean sleep regularity across a two-week period for each participant plotted by age, with fitted GAMM trajectories for FHR (triangles) and CO (circles) while controlling for sex, observation weekday/end, race, past year diagnosis of mood disorder, and actigraph watch type. Shaded regions represent the 95% confidence intervals of the model fits. The p-value corresponds to the global test of the group-by-age interaction, indicating whether overall age-dependent trajectories differ between groups.

(B) Age-varying difference in sleep regularity between familial high-risk and comparison offspring. The solid line is the generalized additive model estimate of the difference. Shaded regions show the simultaneous 95% confidence bands. The dark grey shading identifies the age range in which the confidence band excludes zero, indicating divergence between FHR and CO trajectories.

**Figure S2**

(A) Observed mean sleep regularity across a two-week period for each participant plotted by age, with fitted GAMM trajectories for FHR (triangles) and CO (circles) while controlling for sex, observation weekday/end, race, past year diagnosis of mood disorder, and actigraph watch type. Shaded regions represent the 95% confidence intervals of the model fits. The p-value corresponds to the global test of the group-by-age interaction, indicating whether overall age-dependent trajectories differ between groups.

(B) Age-varying difference in sleep regularity between familial high-risk and comparison offspring. The solid line is the generalized additive model estimate of the difference. Shaded regions show the simultaneous 95% confidence bands. The dark grey shading identifies the age range in which the confidence band excludes zero, indicating divergence between FHR and CO trajectories.

**Figure S3**

(A) Observed mean sleep regularity across a two-week period for each participant plotted by age, with fitted GAMM trajectories for FHR (triangles) and CO (circles) while controlling for sex, observation weekday/end, race, past year diagnosis of mood disorder, and actigraph watch type. Shaded regions represent the 95% confidence intervals of the model fits. The p-value corresponds to the global test of the group-by-age interaction, indicating whether overall age-dependent trajectories differ between groups.

(B) Age-varying difference in sleep regularity between familial high-risk and comparison offspring. The solid line is the generalized additive model estimate of the difference. Shaded regions show the simultaneous 95% confidence bands. The dark grey shading identifies the age range in which the confidence band excludes zero, indicating divergence between FHR and CO trajectories.
